# Mesenchymal Stem Cell-Mediated Effects of Tumor Support or Suppression

**DOI:** 10.3390/ijms161226215

**Published:** 2015-12-16

**Authors:** Ki-Jong Rhee, Jong In Lee, Young Woo Eom

**Affiliations:** 1 Department of Biomedical Laboratory Science, College of Health Sciences, Yonsei University, 1 Yonseidae-gil, Wonju 26493, Korea; kjrhee@yonsei.ac.kr; 2 Department of Hematology-Oncology, Wonju College of Medicine, Yonsei University, 20 Ilsan-ro, Wonju 26426, Korea; oncohem@yonsei.ac.kr; 3 Cell Therapy and Tissue Engineering Center, Wonju College of Medicine, Yonsei University, 20 Ilsan-ro, Wonju 26426, Korea

**Keywords:** mesenchymal stem cells, tumor microenvironment, homing, delivery vehicles

## Abstract

Mesenchymal stem cells (MSCs) can exhibit a marked tropism towards site of tumors. Many studies have reported that tumor progression and metastasis increase by MSCs. In contrast, other studies have shown that MSCs suppress growth of tumors. MSCs contribute to tumor growth promotion by several mechanisms: (1) transition to tumor-associated fibroblasts; (2) suppression of immune response; (3) promotion of angiogenesis; (4) stimulation of epithelial-mesenchymal transition (EMT); (5) contribution to the tumor microenvironment; (6) inhibition of tumor cell apoptosis; and (7) promotion of tumor metastasis. In contrast to the tumor-promoting properties, MSCs inhibit tumor growth by increasing inflammatory infiltration, inhibiting angiogenesis, suppressing Wnt signaling and AKT signaling, and inducing cell cycle arrest and apoptosis. In this review, we will discuss potential mechanisms by which MSC mediates tumor support or suppression and then the possible tumor-specific therapeutic strategies using MSCs as delivery vehicles, based on their homing potential to tumors.

## 1. Introduction

Mesenchymal stem cells (MSCs) are a promising source for cell therapy in regenerative medicine. The therapeutic properties of MSCs are related to their potentials for trans-differentiation, immunomodulation, and trophic factor secretion. The minimal criteria for human MSCs were defined by the International Society for Cellular Therapy, in 2006, as follows: (1) MSCs must be plastic-adherent when maintained under standard culture conditions; (2) more than 95% of cells in a given population of MSCs should express CD105, CD73, and CD90, and lack the expression (less than 2% positive) of CD45, CD34, CD14 or CD11b, CD79α or CD19, and HLA class II surface molecules; (3) MSCs must differentiate into osteoblasts, adipocytes, and chondroblasts under standard conditions *in vitro* [1]. Investigators have isolated MSCs from many different tissues, including bone marrow, adipose tissue, umbilical cord blood, peripheral blood, dermis, liver, skin, and skeletal muscle [2,3,4,5,6,7]. In many studies it has been reported that MSCs that originated in different tissues have similar properties (*i.e.*, expression of cell surface antigens, immunomodulatory capability, and tropism towards tumor) [8,9]. By contrast, it has been reported that different MSCs isolated from two distinct tissues can be recruited into tumor microenvironments, and the different MSC types can be more prompt to transdifferentiate into determined cell types [10]. The different types of MSCs express a distinct set of genes, which is a reflection of its differentiation potential and origin [11,12]. MSCs can be expanded without the loss of their potential for use in clinical applications or differentiation into multiple cell lineages, including adipocytes, osteocytes, chondrocytes, hepatocytes, fibroblasts, and pericytes [13,14,15,16,17]. However, the trans-differentiation of MSCs has rarely been observed in animal models [18]. MSCs can secrete various immunomodulators, such as nitric oxide (NO), prostaglandin (PGE2), indoleamine 2,3-dioxygenase (IDO), interleukin (IL)-6, IL-10, and HLA-G. These soluble factors modulate the function of various immune cells as well as induce T regulatory cells (reviewed in [19]). In addition to the release of immunomodulators, MSCs can directly suppress immune cell activation via cell-to-cell. T-cell proliferation can also by inhibited by MSC by inducing effector T cell apoptosis through interaction of programmed death-1 (PD-1) molecules with its cognate ligands PD-L1 and PD-L2. Furthermore, MSCs can induce T cell anergy by downregulating expression of CD80 and CD86 on antigen-presenting cells [20,21,22]. In addition, MSCs secrete various modulatory factors that can regulate inflammation, cell death, angiogenesis, fibrosis, and tissue regeneration [23]. It has been reported that MSCs secrete trophic factors that promote cell survival (SDF-1, HGF, IGF-1), cell proliferation (EGF, HGF, NGF, TGF-α), and tissue angiogenesis (VEGF) [24,25,26]. Moreover, MSCs can migrate toward injury sites along chemoattractant gradients in the stromal extracellular matrix (ECM) and peripheral blood [27]. In injury sites, MSCs are stimulated by local factors, such as hypoxia, cytokine milieu, and Toll-like receptors ligands. This diverse array of stimuli promotes formation of abundant growth factors that converge to augment tissue regeneration [28,29].

In contrast to the usage of MSCs in regenerative medicine, recent data suggest that MSCs can either augment tumorigenesis or inhibit tumorigenesis [30,31]. In the tumor microenvironment, the tumor attempts to avoid recognition by the immune system while simultaneously secreting inflammatory mediators to establish and maintain a constant state of inflammation [32]. Moreover, the correlation between normal cells, cancer cells, and the matrix within the tumor microenvironments has gained increasing attention, especially because these interactions contribute to certain milestones of cancer, such as immunomodulation, angiogenesis, invasion and metastasis, and apoptotic resistance [33,34]. In several studies, it has been shown that MSCs migrate to the tumor microenvironment and then subsequently support formation of tumor vasculature, enhance the fibrovascular network, and suppress immune reactions, thereby modulating the tumor response to anti-tumor therapy (reviewed in [35,36,37,38,39]). In contrast to their tumor-promoting abilities, MSCs can also suppress tumor growth via inhibition of proliferation-related signaling pathways such as AKT, PI3K, and Wnt, inhibition of cell cycle progression, downregulation of XIAP (X-linked inhibitor of apoptosis protein), and suppression of angiogenesis [40,41,42,43,44,45,46,47].

In this review, we have summarized the mechanisms of MSC-mediated effects of tumor support or suppression and then discussed possible tumor-specific therapeutic strategies using MSCs as delivery vehicles, based on their homing potential to tumors.

## 2. Promotion of Tumor Growth by MSCs

**Figure 1:**
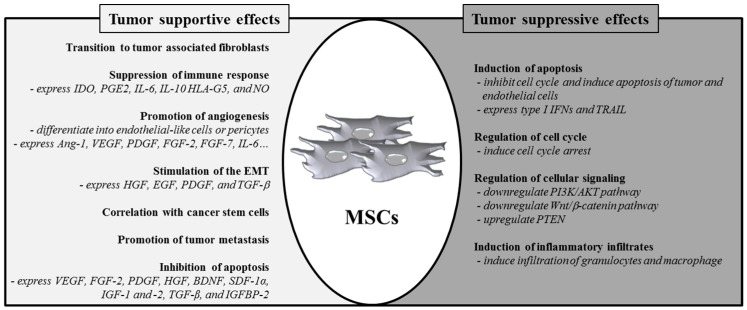
Role of MSCs in tumor formation or suppression. MSCs can regulate transition to tumor-associated fibroblasts, immune response, angiogenesis, EMT, cancer stem cells, metastasis, and apoptosis. Alternatively, growing evidence shows that MSCs inhibit tumor cell function by inducing apoptosis, cell cycle arrest, and inflammatory infiltration and inhibiting the Wnt and AKT signaling pathways.

The tumor microenvironment, which is composed of cancerous cells, non-cancerous cells, and their stroma, influences cancer growth [48]. The tumor stroma harbors many cell types as well as the extracellular matrix. These cells include different kinds of immune cells, fibroblasts, endothelial cells, and myofibroblasts [49]. MSCs move to tumor sites and then incorporate into the tumor stroma [50,51]. These cells interact with each other and with cancer cells, resulting in the promotion of tumor growth. The ability of MSCs to promote tumor growth and metastasis was proved in a breast tumor mouse model [52] and similar results were also obtained from cancer cells co-implanted with MSCs [53,54,55]. Furthermore, allogeneic mice transplanted with B16 melanoma cells did not support tumor formation in the absence of concomitant MSC co-injection [56]. This finding indicates that MSCs exert immunosuppressive effects that were required for tumor initiation. In this review, we will elaborate on how MSCs contribute to tumorigenesis, including (1) by transition to tumor-associated fibroblasts; (2) by suppression of the immune response; (3) by promotion of angiogenesis; (4) by stimulation of epithelial–mesenchymal transition (EMT); (5) through contribution to the tumor microenvironment; (6) by inhibition of tumor cell apoptosis; and (7) by promotion of tumor metastasis (Figure 1).

### 2.1. Transition of Mesenchymal Stem Cells to Tumor-Associated Fibroblasts

Tumors consist of cancerous cells and different stromal cells that form the tumor cellular milieu [34,57]. The tumor stroma consists of an extracellular matrix (ECM) scaffold populated by stromal cells including fibroblasts, immune cells, and endothelial cells. Fibroblasts can be activated in the tumor stroma and activated fibroblasts (also designated myofibroblasts) are called carcinoma-associated fibroblasts (CAFs) or tumor-associated fibroblasts (TAFs). CAFs/TAFs are abundant in most invasive tumors and are composed mainly of cells expressing α-smooth muscle actin (α-SMA) [58]. These cells promote tumor growth and angiogenesis via secretion of stromal-cell derived factor 1 (SDF-1) [59], which binds to CXCR4 expressed by tumor cells [60]. Recently, it was reported that MSCs can differentiate into CAFs/TAFs [37,55,61,62]. Indeed, MSCs can differentiate into myofibroblasts with concomitant increased production of α-SMA, tenascin-C and fibroblast surface protein (FSP), CCL5/RANTES, and SDF-1 [37,61,62,63]. Therefore, the CAFs/TAFs differentiated from MSCs can stimulate tumor growth via the contribution of angiogenesis and the production of tumor-stimulating growth factors.

### 2.2. Suppression of Immune Response in Tumor Microenvironments

In addition to protecting the host against foreign invaders, the immune system recognizes tumor antigens and eliminates malignant tumors [64,65]. Therefore, tumor growth, invasion, and metastasis are important aspects of the tumor immune escape mechanism. During tumor initiation, tumor-associated macrophages (TAMs) and MSCs migrate into tumor microenvironments. TAMs act as the major inflammatory component of the tumor microenvironment [66,67,68] and consist of M1 phenotype cells, which kill pathogens, and M2 phenotype cells, which induce angiogenesis and tissue remodeling [69,70,71]. M2 macrophages can support tumor growth by secreting EGF, PDGF, FGF, VEGF, TGF-β, IL-4, and IL-13 [69,70,72,73,74,75,76]. In contrast, TAMs can show antitumor activities linked to the M1 phenotype via IFN-γ, TNF-α, TGF-β, PGE2, and IL-10 [72,77,78,79,80,81,82]. In addition, M1 TAMs produce oxygen radicals, nitrogen radicals, and pro-inflammatory factors (e.g., IL-1β, IL-6, IL-12, IL-23, and TNF-β) that can facilitate the killing of cancer cells.

In tumor microenvironments, MSCs can be activated by pro-inflammatory cytokines IFN-γ, TNF-α, or IL-1β [52,69,83,84,85], which are secreted by tumor cells and macrophages and then produce immunomodulatory molecules, such as IDO, PGE2, IL-6, IL-10, HLA-G5, and nitric oxide (NO) (reviewed in [86]). IDO is the critical rate-limiting enzyme of tryptophan catabolism through the kynurenine pathway, resulting in tryptophan depletion and halting the growth of various cells. Since both IDO and HLA-G are expressed in the placenta, these molecules likely play a role in immune tolerance during pregnancy. Moreover, IDO and HLA-G inhibit effector T cell proliferation, DC maturation, B cell proliferation, IgG secretion, and nature killer (NK) cell activity [87,88]. PGE2 has a multifunctional role in pathological processes and regulates inflammation and cancer. Production of PGE2 by MSCs is increased following TNF-α or IFN-γ stimulation. Furthermore, PGE2 increases the expression level of anti-inflammatory cytokine IL-10 and decreases expression of TNF-α, IFN-γ, and IL-12 in dendritic cells (DCs) and macrophages [89,90]. PGE2 also dampens secretion of IFN-γ and IL-4 in Th1 and Th2 cells, respectively, and promotes proliferation of Treg cells [19]. MSC-secreted IL-6 inhibited monocyte differentiation toward DCs and subsequently induced a decrease in the stimulatory ability of DCs on T cells [91,92]. In addition, IL-6 secreted by MSCs resulted in the delay of apoptosis of lymphocytes and neutrophils [93,94]. NO is produced by inducible NO synthase (iNOS) via stimulation by inflammatory factors such as IL-1, IFN-γ, and TNF-α [95,96] and also inhibits the functions of T cells [97].

### 2.3. Promotion of Angiogenesis

Blood vessels supply the tumor with nutrients and oxygen, resulting in tumor growth and increased tumor metastasis. Various studies have suggested that MSCs promote tumor angiogenesis through their potential to differentiate into endothelial-like cells or pericytes as well as by their secretion of trophic factors and proangiogenic factors, growth factors, cytokines, and plasminogen activator [55,98,99]. These molecules induce angiogenesis of endothelial cells synergistically [100]. VEGF and FGF-2 are two key vasculogenic factors secreted by MSCs that promote tumor neovascularization [55]. Mobilization and recruitment of MSCs into neovascularization sites can be increased by VEGF expression and then MSCs can be directly differentiated into vascular cells [30,101]. Moreover, VEGF expression by MSCs can be increased by hypoxic conditions, which are common in tumor tissues [102]. In addition, MSCs enhanced blood vessel integrity by inducing expressing of junctional proteins [103]. MSCs also directly increase the angiogenic process as endothelial cells, smooth muscle cells, or pericytes [104]. Rat MSCs that had been incorporated into tumor vessel walls did not express endothelial markers but did express several pericyte markers such as NG2, α-SMA, and PDGFR-β [99]. Taken together, the distinct pericyte marker expression profile of the engrafted MSC as well as the perivascular location suggest that these cells function as pericytes. Additionally, human MSCs co-incubated with conditioned media from a glioblastoma expressed several markers characteristic of pericyte differentiation [105,106].

### 2.4. Epithelial–Mesenchymal Transition (EMT)

Epithelial–mesenchymal transition (EMT) is characterized by downregulation of epithelial cell-associated proteins E-cadherin, γ-catenin/plakoglobin, and ZO-1. In contrast, there is an upregulation of mesenchymal proteins, including N-cadherin, vimentin, fibronectin, and smooth muscle actin [107,108]. While EMT is required for organogenesis and wound healing, epithelial tumor development is also associated with EMT [109]. Accumulating evidence suggests that aberrant EMT promotes tumor invasiveness, tumor metastasis, and drug resistance [110]. In many tumors, molecules such as HGF, EGF, PDGF, and TGF-β are produced from the tumor-associated stroma and act as EMT-inducing signals [109,111,112]. Interestingly, these factors are secreted by MSCs [29] and activate a series of EMT-promoting transcription factors such as Snail, Slug, zinc finger E-box binding homeobox 1, and TWIST [113,114] to transmit EMT-promoting signals [115]. Experimental evidence from a recent study showed that upregulation of EMT-specific genes followed co-culture of breast cancer cells with MSCs expressing decreased levels of an epithelial-specific gene [116]. MSCs also enhanced the metastatic capacity of colon cancer cells with an elevated expression of EMT-associated genes, such as ZEB1, ZEB2, Slug, Snail, and Twist, in a contact-dependent manner. Of note, the EMT-related gene E-cadherin was significantly downregulated [117]. Leptin produced by MSCs enhanced expression of EMT and metastasis genes (SERPINE1, MMP-2, and IL-6) in breast cancer cells. In addition, when SCID/beige mice were co-injected with MCF7 breast cancer cells with MSCs containing leptin shRNA, leptin levels in MSCs decreased and also caused a reduction in MCF7 tumor volume and fewer metastatic lesions in the lungs and livers of mice [118]. Moreover, MSCs can fuse with different cancer cells and show all the classical characteristics of EMT [119,120,121,122,123,124].

### 2.5. Correlation of MSCs with Cancer Stem Cells

Cancer stem cells (CSCs) have been identified in recent years in a variety of solid tumors [125]. There is increasing evidence that these CSCs mediate tumor metastasis and may contribute to relapse following chemotherapy and radiation therapy [126]. Liu *et al.* showed that breast populations of both CSCs and MSCs form a cellular hierarchy in which MSCs expressing aldehyde dehydrogenase regulate breast CSCs through signaling pathways involving IL-6 and CXCL 7 [127]. IL-6 produced by CSCs interacts with IL-6R/gp130 expressed on MSCs, followed by production of CXCL7 by MSCs [127]. In turn, CXCL7 induces the secretion of a number of cytokines from both CSCs and MSCs, including IL-6, IL-8, CXCL6, and CXCL5 [127]. It has been shown that these cytokines trigger proliferation of CSCs and enhance their invasive properties, whereas IL-6 mediates chemotaxis, which promotes MSCs homing to primary tumor growth sites in mouse xenograft models [52,127]. Carcinoma-associated MSCs (CA-MSCs) express BMP2, BMP4, and BMP6. *In vitro* treatment with BMP2 mirrored the effects of CA-MSCs on cancer stem cells while inhibiting BMP signaling, whereas, *in vivo*, BMP2 partially inhibited MSC-promoted tumor growth. These results imply that MSCs can promote tumor growth by increasing the CSC number through BMP expression [128].

### 2.6. Promotion of Tumor Metastasis

Along with the increasing number of cancer metastasis mechanisms being discovered, it has been reported that MSCs can induce metastasis *in vitro* and *in vivo* [52,53,129,130]. When breast cancer cells were co-incubated with human MSCs, the expression of oncogenes and proto-oncogenes was upregulated in breast cancer cells [116]. These molecular changes are accompanied by morphological alterations to a more metastatic phenotype. The breast cancer cells induce secretion of the CCL5 which then induce tumor cell motility, invasiveness, and metastatic potentials [52]. CCL5/RANTES-mediated invasion is also closely related with increased activity of matrix metalloproteinase 9 (MMP-9) [38]. However, this enhanced metastatic ability is reversed when MSCs are injected into separate sites, even if those sites are in close proximity [52]. Other mechanisms such as induction of EMT, regulation of CSCs, and shifting of mesenchymal niches are also involved in tumor metastasis [131].

### 2.7. Inhibition of Apoptosis in Tumor Cells

MSCs can secrete cell regenerative factors continuously, but also secrete factors in response to other various stimuli [132]. Tumor progression is accompanied by hypoxia, starvation, and inflammation. In particular, it was shown that *in vitro* culture of MSCs under hypoxic conditions augmented cellular proliferation. Additionally, the expression of Rex-1 and Oct-4 was increased, leading to the conclusion that MSC stemness was increased during hypoxia [133]. Moreover, under hypoxic and starved conditions, MSCs can survive via autophagy and release many anti-apoptotic or pro-survival factors, such as VEGF, FGF-2, PDGF, HGF, brain-derived neurotropic factor (BDNF), SDF-1α, IGF-1 and IGF-2, transforming growth factor-β (TGF-β), and IGF binding protein-2 (IGFBP-2) [28,134,135,136,137,138]. These factors inhibit tumor cell apoptosis and promote tumor proliferation, while normal MSCs do not take on these properties. In addition to the mitogenic properties of growth factors secreted by MSCs, VEGF and FGF-2 can mediate the expression of Bcl-2, resulting in delaying apoptosis [139,140,141], while indirect angiogenic factors can induce the expression of VEGF and FGF-2 [142]. Moreover, SDF-1α was reported to prevent drug-induced apoptosis of chronic lymphocytic leukemia (CLL) cells [143]. Furthermore, it has been reported that VEGF, FGF-2, HGF, and IGF-1 expressed by MSCs stimulate the angiogenic and anti-apoptotic effects after hypoxic conditioning [28,137]. Although little is known as to how MSCs under hypoxic conditions exert supportive effects on tumor cells directly, MSC-secreted growth factors stimulated by hypoxia can endow tumor supportive effects in the tumor microenvironment through angiogenic and anti-apoptotic effects.

## 3. Suppression of Tumor Growth by MSCs

Although many studies have shown that MSCs have tumor-promoting properties, many other studies have shown that MSCs have tumor-suppressive properties (Figure 1) (reviewed in [35]). In this regard, MSCs are thought to suppress tumor growth by increasing infiltration of inflammatory cells [144], inhibiting angiogenesis [47], suppressing the signaling of Wnt and AKT [40,41,42], and inducing cell cycle arrest and apoptosis [44,45,46,145]. Recently, Ryu *et al.* reported that when MSCs derived from adipose tissue was grown at high cell density, they synthesized IFN-β, which then suppressed the growth of MCF-7 cells [146]. Moreover, MSCs primed with IFN-γ or cultured with tri-dimensional systems can express TRAIL, which induces tumor cell-specific apoptosis [132,147].

### 3.1. Induction of Apoptosis of Cancer Cells and Endothelial Cells

Lu *et al.* demonstrated that MSCs had an inhibitory effect on mouse tumor cells and ascitogenic hepatoma cells in a cell-dependent manner through the caspase 3 pathway [145]. They also found that MSCs increased mRNA expression of p21, a negative regulator of cell cycle. Those data strongly implied that MSCs exerted tumor inhibitory effects in the absence of host immunosuppression, by inducing G0/G1 phase arrest and apoptosis of cancer cells [145]. In SCID mice xenografted with disseminated non-Hodgkin’s lymphoma, MSCs displayed anti-cancer activity [148]. A single injection of MSC increased the survival of animals with aggressive lymphomas. It was also reported that a significant induction of endothelial cell apoptosis occurred in a direct co-culture of MSCs with endothelial cells, suggesting that MSCs exerted anti-angiogenic activity through endothelial cell apoptosis [148]. These findings were consistent with the results from studies that demonstrated that MSCs exhibited a potent anti-angiogenic activity in Kaposi sarcomas with high vascularity and in *in vitro* endothelial cell cultures [40,47]. Moreover, Dasari *et al.* reported that downregulation of the anti-apoptotic inhibitor, X-linked inhibitor of apoptosis protein (XIAP), by human umbilical cord blood-derived mesenchymal stem cell (hUCBSC) treatment induced apoptosis of glioma cells and xenograft cells through the activation of caspase-3 and caspase-9 [45,46]. Recently, MSCs cultured at a high density expressed type I IFN, leading to the cell death of breast cancer cells, MCF-7, and MDR-MB-231 cells [146]. Moreover, MSCs primed with IFN-γ or cultured with tri-dimensional systems can express TRAIL, which induces tumor cell-specific apoptosis [132,147].

### 3.2. Regulation of Cell Cycle

MSCs secrete a variety of cytokines that induce cell cycle arrest of tumor cells, albeit transiently, at the G1 phase through expression of CyclinA, CyclinE, CyclinD2, and p27KIP1 [44,145,149,150,151,152,153]. Human stromal cells that were differentiated from adipose tissue (ADSC) and ADSC-conditioned cell culture medium suppressed tumors [145]. Furthermore, the ADSC-conditioned cell culture medium stimulated necrosis of cancer cells after G1-phase arrest in the absence of apoptosis. Finally, when ADSC was introduced into pancreatic adenocarcinoma, the tumor did not grow [145]. Similarly, tumor cells that were cultivated with MSCs *in vitro* also were arrested at the G1 phase [153]. However, when non-obese diabetic-severe combined immunodeficient mice were injected with MSCs and tumor cells, their growth was more augmented compared to tumor cell injection alone. Although it has been reported that MSCs can induce cell cycle arrest of tumor cells *in vitro*, little is known about the exact mechanisms. In our experiment, cell cycle retardation or arrest can be induced in certain tumor cell types and under certain co-culture conditions (type of media, cell concentration or co-culture time). While we cannot explain the exact mechanism(s), a number of studies by different groups, including ours, have shown that tumor cell cycle arrest does occur.

### 3.3. Regulation of Cellular Signaling

The phosphoinositide 3-kinase (PI3K)/AKT and WNT/β-catenin signaling pathway controls cell survival, proliferation, growth, migration, and metabolism [154,155,156,157,158,159]. In tumor biology, numerous studies describe the requirement of AKT signaling for the migration, invasion, and survival of tumor cells. The WNT signaling pathway has also been associated with the development of carcinomas of the breast, liver, colon, skin, stomach, and ovary [160,161,162,163,164,165,166,167,168,169,170,171]. In a Kaposi’s sarcoma model, intravenously injected MSCs migrated to tumors and effectively inhibited tumor proliferation through inhibition of AKT [40]. Moreover, in glioma cells, PTEN was upregulated by hUCBSCs, resulting in AKT downregulation [45]. In addition to inhibition of the PI3K/AKT signaling pathway, MSCs can also suppress the WNT/β-catenin pathway through induced expression of DKK-1 [41,42,43]. These findings showed that β-catenin was downregulated in human carcinoma cell lines (hepatocellular, H7402 and HepG2; breast, MCF-7; hematopoietic, K562 and HL60) by DKK-1 secreted from MSCs. When DKK-1 activity was inhibited by either using neutralizing anti-DKK-1 antibodies or RNAi, an attenuation of the inhibitory effects of MSCs on tumor cell proliferation was observed [41,42,43].

### 3.4. Induction of Inflammatory Infiltrates

Although MSCs can suppress immune responses, Ohlsson *et al.* reported that co-administration of tumor cells and MSCs caused increased infiltration of granulocytes and monocytes than did separate treatments with tumor cells or MSCs alone *in vivo* [144]. They used a preformed gelatin matrix incorporating rat colon cancer cells and/or MSCs, which were then surgically transplanted subcutaneously into rats to monitor tumor outgrowth and the ensuing inflammatory response. MSCs inhibited rat colon carcinoma [144]. The increased infiltrations of both granulocytes and macrophages were noted to be much higher in rats co-injected with tumors and MSCs than in rats injected with tumors without MSCs. These data suggested that MSCs had pro-inflammatory effects in this model, although expression of MHC-class I was low and MHC-class II was absent in MSCs. In fact, the researchers observed that an increased degree of infiltration of granulocytes and macrophages was also seen, but to a lesser extent, when only MSCs were added to the gelatin [144].

## 4. MSCs as Delivery Vehicles for Pro-Apoptotic Agents

Regardless of delivery routes such as intravenous [40,50,172,173,174,175], intraperitoneal [176], or intracerebral [173], MSCs have the ability to migrate and infiltrate into the tumor microenvironment [177,178,179,180,181]. However, it is known that systemic administration of MSCs into the vascular system result in accumulation of MSCs in the lung in large numbers [182,183]. Moreover, in the case of MSC therapy for liver failure, depending on the route of injection and the status of the liver, MSCs can differentiate into myofibroblasts [184,185]. Thus, the route of injection and the optimal therapeutic timing according to disease status, including tumor status, must be considered to reduce the side effects of MSCs. Janowski *et al.* reported that cell number and infusion velocity are critical factors in developing safe protocols for stem cell transplantation [186]. Furthermore, Schrepfer *et al.* demonstrated that pretreatment with intravenous sodium nitroprusside can decrease cell trapping in the lungs [187].

Based on this capability of homing to the tumor, MSCs can be utilized to deliver pro-apoptotic agents straight into the tumor microenvironment. Numerous studies have used MSCs engineered to express and deliver a variety of anti-cancer drugs, such as type I interferon (IFN-α and IFN-β), IL-12, IL-2, CXCL1, oncolytic virus, cytokine deaminase, nanoparticles, and TRAIL [50,172,173,176,188,189,190,191,192,193,194,195,196,197,198,199,200,201,202]. IL-2 over-expressing MSCs improve immune surveillance against glioma [192] and melanoma [203] and reduce metastasis from a subcutaneous model [193]. CXCL1 and IL-12 expressed by MSCs activate both T cells and NK cells, and result in a substantial decrease in lung and breast tumors and melanoma [175,191,193,195,204]. Because the local concentrations of IFN-α, IFN-β, and TRAIL can be increased by genetically engineered MSCs, the activities of IFN-α, IFN-β, and TRAIL on suppression of tumor mass and animal survival are potentiated more effectively than are the activities of IFN-α, IFN-β and TRAIL used in a systematical treatment [50,173,188,189,190]. MSCs have also been engineered to deliver conditional replicative oncolytic viruses, which selectively target and inhibit tumor cells without affecting normal cells, to diminish tumor growth and metastasis [176,196,197,205]. Furthermore, MSCs can be used to increase targeting efficiency of nanoparticulate drug delivery system-based cancer therapies [200], and to enhance the cytotoxicity of pro-drugs by having a converting enzyme expressed in MSCs at the site of a tumor [198,199,206,207,208,209].

## 5. Conclusions

Although MSC therapy in regenerative medicine is considered feasible and safe in patients [210], the ability of MSCs to promote tumorigenesis of pre-existing tumors must be scrutinized. This review highlights the mechanisms of MSC-mediated effects of tumor support or suppression and then discusses the possible modulation of MSCs for tumor therapy. MSCs demonstrate a tropism for tumors and then infiltrate the tumor stroma [50,51]. These cells interact with each other and with cancer cells, resulting in promotion of tumor growth. MSCs contribute to tumor growth promotion in several mechanisms, including (1) by transition to tumor associated fibroblasts; (2) by suppression of the immune response; (3) by promotion of angiogenesis; (4) by stimulation of the epithelial–mesenchymal transition (EMT); (5) through contribution to the tumor microenvironment; (6) by inhibition of tumor cell apoptosis; and (7) via promotion of tumor metastasis. In contrast to the tumor-augmenting characteristics, many studies have reported that MSCs can prevent growth of tumors [35]. MSCs inhibit tumor growth by increasing inflammatory infiltration [144], inhibiting angiogenesis [47], suppressing the signaling of Wnt [41,42] and AKT [40], and inducing cell cycle arrest and apoptosis [44,45,46,145]. In spite of these tumor-suppressive mechanisms, not much is understood regarding the molecular mechanisms by which MSCs inhibit tumor cells because it has not yet been shown which ligand induces the suppression of tumor growth. MSCs and tumor cells interact in a myriad of ways and thus the MSCs can either support or suppress tumor growth depending on multiple factors. Myriad cell types can influence tumor progression. Moreover, the timing of MSC introduction into the tumor microenvironment may be critical to elucidate the dual role of MSCs in tumor support or suppression. In several studies, tumor growth inhibition was observed when MSCs were introduced into established tumors and when direct contact of MSCs and tumor was suppressed by gelatin matrix or intravenous delivery of MSCs during tumor initiation [40,144]. By contrast, many studies have demonstrated that the tumor was promoted when MSCs and tumor cells were injected simultaneously [52,53,54,55,56]. Based on a marked tropism of MSCs for tumors, MSCs can be applied to deliver pro-apoptotic agents directly into the tumor microenvironment for tumor therapy. More robust studies characterizing the mechanisms of tumor support or suppression by MSCs may increase the utilization of MSCs in regenerative medicine without risk of promoting pre-existing tumor cell growth and advance the possibility of developing therapeutic strategies using MSCs with minimal side effects.
